# Highly-resistant *E. coli* as a Common Cause of Paediatric Diarrhoea in India

**DOI:** 10.3329/jhpn.v31i3.16835

**Published:** 2013-09

**Authors:** Prabhav Aggarwal, Beena Uppal, Roumi Ghosh, S. Krishna Prakash, Krishnan Rajeshwari

**Affiliations:** ^1^Department of Microbiology, Maulana Azad Medical College, New Delhi, India;; ^2^Department of Pediatrics, Maulana Azad Medical College and associated Lok Nayak Hospital, New Delhi, India

Sir,

It is well-documented that diarrhoea is one of the major causes of morbidity and mortality among children. UNICEF and WHO have ranked it the second-most common cause of death following closely behind pneumonia in children of aged less than five years. They also noted that India has more number of deaths (estimated 386,600 annually) due to diarrhoea than any other country in the world (1). Common causes of bacterial diarrhoea include diarrhoeagenic *Escherichia coli* (DEC), *Shigella* species, *Vibrio cholerae, Salmonella* species, *Campylobacter*, and *Yersinia enterocolitica*.

UNICEF and WHO recommend the use of new formula of ORS for preventing and treating dehydration associated with paediatric diarrhoea (1). However, in our experience, it is common to prescribe antimicrobial agents to children with more severe diarrhoea and those who do not respond adequately to rehydration therapy. Rapidly-growing antimicrobial resistance is a global concern, leaving the physicians with very few choices for antimicrobials. The situation has only been worsened by production of extended-spectrum beta-lactamase (ESBL) which renders the bacteria resistant to the penicillins, first-, second-, and third-generation cephalosporins, and aztreonam (but not the cephamycins or carbapenems) by hydrolysis of these antibiotics (2).

A study was undertaken with the aim of determining the role of *E. coli* in acute diarrhoea among children aged less than five years. It was also aimed at determining the degree of antimicrobial resistance and production of ESBL by the diarrhoeagenic *E. coli* (DEC).

The study was conducted over a period of one-and-a half years from 1 October 2010 to 31 March 2012 at a tertiary-care centre in New Delhi. All children aged less than five years presenting during this period with acute diarrhoea (with or without visible blood/mucus) of duration less than 14 days were included in the study. In total, stool samples were collected from 347 children in clean plastic containers and transported to microbiology laboratory within two hours after collection. In case of delay of more than two hours, samples were transported in Cary-Blair medium/buffered glycerol saline, or stored at 4 °C. Wet mounts were examined for presence of pus cells, RBC, and parasitic ova/cysts or trophozoites. Stool samples were cultured on MacConkey agar, Xylose desoxycholate agar (XLD), *Salmonella-Shigella* agar, and bile salt agar (BSA). The stool samples were also inoculated in Selenite F broth and alkaline peptone water (APW) for enrichment. The plates were incubated at 37 °C for 18-24 hours. The organisms were identified on the basis of colony characteristics and biochemical reactions. *E. coli* isolates were subjected to serotyping by the slide agglutination test (Denka Seiken Co., Ltd., Tokyo, Japan).

Bacterial enteropathogens were subjected to antimicrobial susceptibility testing, using disc diffusion method (3) against a wide range of antimicrobial agents and phenotypic confirmatory test for ESBL production according to the CLSI guidelines (2011) (3). ATCC25922 was used as the control strain. Decreased susceptibility to ceftazidime (zone diameter ≤22 mm) and/or cefotaxime (zone diameter ≤27 mm) encountered was used as initial screening test for ESBL production. These isolates were subjected to the phenotypic confirmatory test (3) by the combination disc method, utilizing (i) ceftazidime (30 µg) and ceftazidime/clavulanic acid (30 µg+10 µg) and (ii) cefotaxime (30 µg) and cefotaxime/clavulanic acid (30 µg+10 µg) (HiMedia, Mumbai, India). An increase in zone diameter of one or both antibiotics by ≥5 mm was taken as indicative of ESBL production.

Of the stool specimens from 347 children, pathogenic organisms were isolated from 156 (44.9%) patients. Diarrhoeagenic *E. coli* was the most common bacterial agent, responsible for 87 (25.1%) cases. By serotyping, Enteropathogenic *E. coli* was found to be the most frequent type among *E. coli* specimens (48, 55.1%), followed by Enterotoxigenic *E. coli* (29, 33.3%), Enteroaggregative *E. coli* (9, 10.3%), and Enterohaemorrhagic *E. coli* (1, 1.1%). *Shigella* spp. and *Vibrio cholerae* were isolated in 36 (10.4%) and 13 (3.7%) cases respectively. Parasites were identified in 20 patients including 11 (3.25%) *Giardia*, 5 (1.4%) *Ascaris lumbricoides*, and 4 (1.2%) *Entamoeba hisolytica.*

A large number of these strains were found to show multidrug resistance to three or more classes of antimicrobials (72.4%). All DEC strains were resistant to nalidixic acid (100%) while more than two-thirds were resistant to ampicillin (90.8%), doxycyline (80.5%), cotrimoxazole (95.8%), ofloxacin (73.3%), and ciprofloxacin (72.4%) (Table 1). It was further seen that 68 (78.1%) of the 87 DEC strains were resistant to at least one of the third-generation cephalosporin tested (ceftriaxone, cefotaxime, and ceftazidime). These isolates were considered to be screening test-positive for ESBL production (3). These were then subjected to phenotypic confirmatory test (3), and 56 (64.3%) of DEC isolates were confirmed to be ESBL producers (Table 2). Therefore, among the antimicrobials tested, only aminoglycosides, carbapenems, and tigecycline were found to be effective drugs. Resistance levels among various groups of DEC (EPEC, ETEC, EAEC, and EHEC) were similar to one another (Table 1).

**Table 1.** Antimicrobial resistance of diarrhoeagenic *E. coli* (%)

	Diarrhoeagenic *E. coli* (n=87)	EPEC (n=48)	ETEC (n=29)	EAEC (n=9)	EHEC (n=1)
Ampicillin	90.8	91.7	89.7	88.9	100
Ampicillin/Sulbactam	65.5	66.7	65.5	56.7	100
Cotrimoxazole	75.8	75	75.9	78.8	100
Amikacin	3.4	2.1	3.4	0	100
Gentamicin	27.5	22.9	34.4	22.2	100
Doxycycline	80.5	83.3	72.4	77.8	100
Nalidixic acid	100	100	100	100	100
Ciprofloxacin	72.4	70.8	79.3	55.6	100
Ofloxacin	73.3	70.8	82.7	66.7	100
Ceftriaxone	73.0	70.8	79.3	66.7	100
Cefotaxime	78.1	77.1	79.3	77.8	100
Ceftazidime	74.1	72.9	75.9	77.8	100
Imipenem	1.1	2.1	0	0	0
Meropenem	1.1	2.1	0	0	0
Ertapenem	1.1	2.1	0	0	0
Tigecycline	0	0	0	0	0

EAEC=Enteroaggregative *Escherichia coli*;

EHEC=Enterohaemorrhagic *Escherichia coli;*

EPEC=Enteropatho-genic *Escherichia coli*;

ETEC=Enterotoxigenic *Escherichia coli*

In our study, DEC was the most frequent cause of bacterial diarrhoea in children aged less than five years. Similar findings were observed by Nair *et al*. who reported DEC (19.4%) to be the most common cause in the same age-group, followed by *Vibrio cholerae* (17%) (4) in a study from Kolkata, India. A study from Peru also reported DEC to be the most frequent offender among infants (29%). However, *Shigella* spp. or *Vibrio cholerae* were not isolated in their study (5). These differences may be explained on the basis of geographical variations.

**Table 2.** Results of test for production of ESBL

	Cefotaxime/Cefotaxime+Clavulanic acid	Ceftazidime/Ceftazidime+Clavulanic acid
Screening test-positive (n=87)	68 (78.1%)	65 (74.7%)
Phenotypic confirmatory test-positive (n=87)	56 (64.3%)	53 (60.9%)
ESBL=Extended-sepectrum beta-lactamase		

Similar to our finding, Lanjewar *et al*. found 100% resistance to nalidixic acid in a study conducted in India. However, resistance to fluoroquinilones (86.05%) and cotrimoxazole (90.60%) was higher, and the resistance to cefotaxime was lower (51.16%) (6). On the other hand, studies from developed countries have reported a lower degree of resistance of DEC to various antimicrobial agents. A study from Peru reported high resistance to ampicillin (85%), cotrimaxazole (79%), and tetracycline (65%). However, they did not report any strain showing resistance to the third-generation cephalosporins (5). Pérez *et al*. also reported much lower resistance compared to our study, with only 40% of the isolates showing resistance to ampicillin, less than 1% to third-generation cephalosporins and none to fluoroquinolones (7) in a study from Costa Rica.

A high level of ESBL production is a matter of concern for all as these substantially decrease the effectiveness of the third-generation cephalosporins. These were often regarded as reserve drugs, to be used only when resistance to other antimicrobials is seen. Studies on ESBL production by diarrhoeagenic *E. coli* are limited. However, the Study for Monitoring Antimicrobial Resistance Trends (SMART) Program 2007 showed similar levels of ESBL production by *E. coli* isolated from a different site (intra-abdominal infections) (8). It also mentioned that the levels were the highest in India (79%) while Australia had the lowest levels (7.7%). Now, with the acquisition of bla_NDM-1_ by several Gram-negative organisms, a high level of resistance to carbapenems may soon develop (9). The risk is real, and the situation is grave.

## Limitations

An important limitation of our study was that the designation of DEC was based on serotyping. Although serotyping is used frequently for identification of the diarrhoeagenic strains, not all isolates identified by serotyping are enterovirulent, and pathogenicity may not be limited to particular serogroups or serovars (10). Hence, all the *E. coli* strains that were identified as diarrhoeagenic may not be responsible for the symptoms in the children. Nevertheless, the presence of highly-resistant *E. coli* in the gut represents a problem, not only in the form of treatment of the current infection but may also serve as source of resistance genes to other pathogenic bacteria present in the gut.

## Conclusions

We conclude that DEC are the most common bacterial cause of paediatric diarrhoea in our region with majority of the strains being resistant to several antimicrobials. These findings suggest that the use of antimicrobial agents for treatment of diarrhoea should be regulated more stringently and restricted to the more serious cases of bacterial diarrhoea. Over-the-counter availability of these drugs and self-medication by the patients need to be checked. Immediate measures are required to halt the development of further resistance and gradually reverse it. The task force of the Indian Ministry of Health and Family Welfare has announced a new national antimicrobial policy in 2011 to look into these matters (11). We hope these guidelines will achieve their goals.
